# Early-pregnancy serum iron as a nutrition-related clinical laboratory indicator for preeclampsia risk stratification: a retrospective cohort study

**DOI:** 10.3389/fnut.2026.1853277

**Published:** 2026-06-10

**Authors:** Sijia Fang, Qianlan Zhang, Ying Chen, Chaoyan Yue

**Affiliations:** 1 Shanghai Jiai Genetic and IVF Institute, Obstetrics and Gynecology Hospital of Fudan University, Shanghai, China; 2 Obstetrics & Gynecology Hospital of Fudan University, Shanghai Key Lab of Reproduction and Development, Shanghai Key Lab of Female Reproductive Endocrine Related Diseases, Shanghai, China

**Keywords:** nutrition-related clinical laboratory indicators, precision medicine, preeclampsia, pregnancy outcomes, serum iron

## Abstract

**Background:**

Nutrition-related clinical laboratory indicators are valuable for risk assessment, early identification, and precision management in obstetric and gynecologic diseases. Serum iron, a commonly used laboratory marker reflecting iron metabolism status, has shown inconsistent associations with preeclampsia (PE).

**Objective:**

To investigate the association between early-pregnancy serum iron levels and the risk of PE, and to evaluate their associations with low birth weight, cesarean delivery, and preterm birth.

**Methods:**

This single-center retrospective cohort study included 37,643 pregnant women. Early-pregnancy serum iron level was the exposure variable, PE the primary outcome, and low birth weight, cesarean delivery, and preterm birth secondary outcomes. Logistic regression models (non-adjusted, Adjust I, and Adjust II) assessed associations between serum iron and each outcome. Smooth curve fitting illustrated the relationship between serum iron and PE risk. Stratified analyses by age, BMI, and parity, along with interaction analysis, evaluated the robustness of the association.

**Results:**

Serum iron levels were lower in the PE group than in the non-PE group (20.45 ± 6.16 vs. 21.32 ± 6.11 μmol/L, *p* < 0.001). Curve fitting, adjusted for age, BMI, and hemoglobin, showed that PE risk generally decreased with increasing serum iron. In the non-adjusted model, each 10 μmol/L increase in serum iron was associated with a 21% reduction in PE risk (OR = 0.79, 95% CI: 0.73–0.85, *p* < 0.0001); In the fully adjusted model, each 10 μmol/L increase in serum iron was associated with a lower risk of PE (OR = 0.89, 95% CI: 0.82–0.97, *p* = 0.0071). For other outcomes, higher serum iron was significantly associated with lower risk of cesarean delivery, whereas no stable significant associations were observed for low birth weight or preterm birth. Stratified analyses showed the inverse association between serum iron and PE was more evident among women aged <35 years, those with BMI < 24 kg/m^2^, and primiparous women; however, interaction tests for age, BMI, and parity were not statistically significant.

**Conclusion:**

Higher early-pregnancy serum iron levels were significantly associated with lower risk of PE, although its clinical utility still requires further validation in prospective studies.

## 1 Introduction

Preeclampsia (PE) is one of the major hypertensive complications during pregnancy, typically occurring after 20 weeks of gestation and characterized by new-onset hypertension accompanied by proteinuria and/or maternal organ dysfunction and placental abnormalities (1–5). PE not only significantly increases maternal and perinatal morbidity and mortality, but is also associated with fetal growth restriction, preterm birth, and long-term maternal cardiovascular and metabolic abnormalities (1–5). Therefore, establishing earlier, more accessible, and more individualized risk identification strategies remains an important clinical issue in the era of precision medicine in obstetrics (1–5).

Iron is one of the most critical trace nutrients during pregnancy and is involved in erythropoiesis, placental development, oxygen transport, and maternal-fetal iron homeostasis regulation (6–14). In recent years, studies on iron deficiency, iron deficiency anemia, iron screening, and iron supplementation during pregnancy have continued to increase. However, current evidence has mainly focused on anemia and traditional perinatal outcomes, while the application of different iron status indicators in disease prediction and precision management still faces challenges, including lack of standardization, insufficient threshold definition, and limited clinical generalizability (6–13). As a readily available and low-cost laboratory indicator in clinical practice, whether serum iron levels in early pregnancy are associated with the subsequent risk of obstetric and gynecologic diseases, especially PE, still warrants further investigation (6–13).

At present, no consensus has been reached regarding the relationship between iron status and PE (15–24). Some studies have suggested that women with PE may present with higher serum iron, ferritin, or hepcidin levels at the time of disease onset or during the second and third trimesters (15, 17, 23–25), whereas other studies have not observed consistent differences, or have suggested that lower serum iron in early pregnancy is associated with an increased risk of subsequent hypertensive disorders of pregnancy (16, 18, 19). In addition, findings from Mendelian randomization studies have also been inconsistent (20–22). These discrepancies may be related to differences in the timing of measurement, the type of iron status indicators assessed, study design, population characteristics, and inflammatory status or iron supplementation (15–24). Therefore, based on a large cohort of pregnant women, this study aimed to investigate the association between early-pregnancy serum iron levels and the risk of PE, and further evaluate its associations with other adverse pregnancy outcomes, in order to provide evidence for the application of nutrition-related laboratory indicators in the precision management of obstetric and gynecologic diseases (15–24).

## 2 Materials and methods

### 2.1 Study design and study population

This was a single-center retrospective cohort study. The study population was derived from pregnant women who underwent prenatal examinations and completed early-pregnancy laboratory testing at the Obstetrics and Gynecology Hospital of Fudan University between 2018 and 2024. The inclusion criteria were singleton pregnancy, completion of serum iron testing in early pregnancy, and availability of pregnancy outcome data. Clinical covariates, including maternal demographic characteristics, blood pressure, laboratory measurements, lifestyle factors, and pregnancy-related information, were extracted from the electronic medical record system. The exclusion criteria were multiple pregnancy and missing data on key exposure or outcome variables. A total of 37,643 pregnant women were ultimately included in the analysis. The study was approved by the Ethics Committee of Fudan University. Serum iron measurement was part of the routine first-trimester prenatal laboratory panel offered to all pregnant women at our hospital.

### 2.2 Exposure variable and covariates

Early-pregnancy serum iron level was defined as the main exposure variable. Serum iron was entered into the models as a continuous variable, and the corresponding odds ratios (ORs) and 95% confidence intervals (95% CIs) were calculated for each 10 μmol/L increase. Serum iron was measured using a Hitachi Automatic Analyzer (Hitachi High-Tech Corporation, Tokyo, Japan) with reagents from DiaSys Diagnostic Systems GmbH (Holzheim, Germany).

Covariates adjusted for in the models included age, parity, BMI, systolic blood pressure, diastolic blood pressure, family history of hypertension, family history of diabetes, smoking, alcohol consumption, *in vitro* fertilization-embryo transfer (IVF), gestational week at testing, albumin (ALB), and hemoglobin (HGB). Blood pressure values used in the adjusted models were those recorded at the establishment of the prenatal care record, which corresponded temporally to the first-trimester serum iron measurement; therefore, these values reflect blood pressure levels in early pregnancy rather than pre-pregnancy blood pressure. Smoking and alcohol consumption variables were also derived from medical history documented at the same visit and primarily represent pre-pregnancy smoking and drinking status. The Adjust I model was adjusted for age, BMI, and HGB. The Adjust II model was further adjusted for parity, systolic blood pressure, diastolic blood pressure, family history of hypertension, family history of diabetes, smoking, alcohol consumption, IVF, gestational week at testing, ALB, and HGB.

### 2.3 Outcome definition

The primary outcome was PE. PE was defined according to the ACOG Practice Bulletin No. 222 criteria and identified from clinical diagnostic records (2). Secondary outcomes included low birth weight, cesarean delivery, and preterm birth. Low birth weight was defined as birth weight <2,500 g; cesarean delivery was determined according to the recorded mode of delivery; and preterm birth was defined as delivery before 37 weeks of gestation.

### 2.4 Statistical analysis

Continuous variables were expressed as mean ± standard deviation, and categorical variables were expressed as number (percentage). For group comparisons, normally distributed continuous variables were analyzed using independent samples t-tests, markedly skewed continuous variables were analyzed using the Wilcoxon rank-sum test, and categorical variables were compared using the χ^2^ test. Logistic regression models were used to evaluate the associations between serum iron and PE as well as other pregnancy outcomes, with results presented as ORs and 95% CIs. Three models were constructed: a non-adjusted model, an Adjust I model, and an Adjust II model. Adjust II model served as the primary analysis model for deriving the final effect estimates.

To further evaluate the dose–response relationship between serum iron and PE, smooth curve fitting based on a generalized additive model was performed using the Adjust I model. Serum iron was plotted on the x-axis and the predicted probability of PE on the y-axis, with the fitted curve and its 95% CI presented to visually assess the trend in PE risk across serum iron levels.

Stratified analyses were performed according to age (<35 years vs. ≥35 years), BMI (<24 kg/m^2^ vs. ≥24 kg/m^2^), and parity (primiparous vs. multiparous) to assess the robustness of the association between serum iron and PE across different subgroups. Interaction analysis was conducted to evaluate effect modification, and a P for interaction >0.05 indicated no evidence of effect modification. All statistical tests were two-sided, and *p* < 0.05 was considered statistically significant. Statistical analyses were performed using R version 3.5.1.

## 3 Results

### 3.1 Baseline characteristics of the study population

A total of 37,643 pregnant women were included in this study, among whom 2,218 developed preeclampsia (PE), corresponding to an incidence of 5.89%. As shown in Table 1, the PE group had lower serum iron levels than the non-PE group. Because this retrospective study was based on routinely collected clinical data, the number of available observations differed across outcomes. The sample sizes included in the analyses were 37,643 for preeclampsia, 37,624 for low birth weight, 22,674 for cesarean delivery, and 24,505 for preterm birth. Analyses were conducted using available data for each corresponding outcome. The overall prevalence of anemia, defined as hemoglobin <110 g/L, was 3.17%.

**Table 1:** Baseline characteristics of participants.

Characteristic	Non-PE (*n* = 35,425)	PE (*n* = 2,218)	*p*-value
Age, years	31.34 ± 3.96	32.01 ± 4.36	<0.001
BMI, kg/m^2^	21.31 ± 2.94	23.48 ± 4.00	<0.001
Systolic blood pressure, mmHg	114.59 ± 12.02	124.70 ± 14.47	<0.001
Diastolic blood pressure, mmHg	69.25 ± 9.42	77.18 ± 10.97	<0.001
Gestational week at testing	10.75 ± 2.27	10.86 ± 2.34	0.019
Albumin, g/L	43.22 ± 2.55	43.22 ± 2.69	0.956
Hemoglobin, g/L	126.49 ± 9.38	129.65 ± 9.76	<0.001
Serum iron, μmol/L	21.32 ± 6.11	20.45 ± 6.16	<0.001
Parity			<0.001
Primiparous	26,505 (76.05%)	1,792 (82.85%)	
Multiparous	8,346 (23.95%)	371 (17.15%)	
Family history of hypertension	5,184 (14.64%)	552 (24.89%)	<0.001
Family history of diabetes	2,159 (6.10%)	180 (8.12%)	<0.001
Tobacco use	634 (1.79%)	43 (1.94%)	0.609
Alcohol use	1,458 (4.12%)	56 (2.52%)	<0.001
IVF	1,950 (5.50%)	268 (12.08%)	<0.001
Low birth weight	1,362 (3.85%)	252 (11.37%)	<0.001
Cesarean delivery	10,793 (51.30%)	1,188 (72.66%)	<0.001
Preterm birth	1,621 (7.04%)	320 (21.45%)	<0.001

### 3.2 Associations of serum iron with PE and other adverse pregnancy outcomes

**Figure 1:**
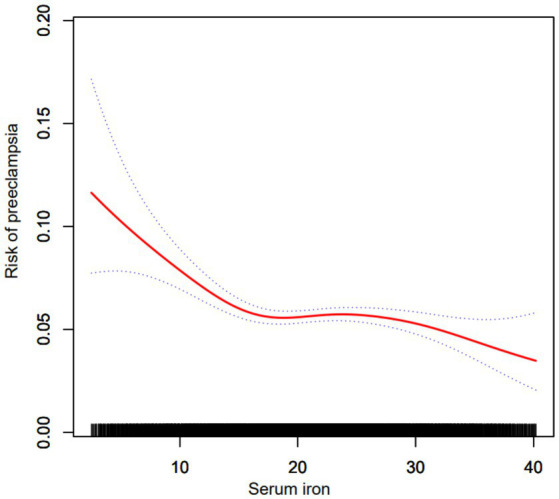
The association between serum iron and the risk of preeclampsia. The red solid line represents the fitted curve, and the blue dashed lines represent the 95% confidence intervals. Fitted curve was adjusted for age, BMI, and hemoglobin.

The fitted curve is shown in Figure 1. After adjustment for age, BMI, and hemoglobin, the risk of PE generally decreased with increasing serum iron levels. As shown in Table 2, higher serum iron levels were significantly associated with a lower risk of PE. In the unadjusted model, each 10 μmol/L increase in serum iron was associated with a 21% reduction in the risk of PE. In the Adjust I model, after adjustment for age, BMI, and hemoglobin, this association remained significant. In the fully adjusted model (adjust II), which incorporated parity, systolic blood pressure, diastolic blood pressure, family history of hypertension, family history of diabetes, smoking, alcohol consumption, IVF, gestational week at testing, albumin, and hemoglobin and served as the primary analysis, the inverse association between serum iron and PE risk remained significant. For other pregnancy outcomes, higher serum iron levels were significantly associated with a lower risk of cesarean delivery, and this association remained statistically significant across all models. No stable significant associations were observed between serum iron and low birth weight or preterm birth.

**Table 2:** Association of serum iron levels with preeclampsia and adverse pregnancy outcomes.

Outcome	Non-adjusted OR (95% CI)	*P*-value	Adjusted I OR (95% CI)	*P*-value	Adjusted II OR (95% CI)	*P*-value
Preeclampsia	0.79 (0.73, 0.85)	<0.0001	0.84 (0.77, 0.91)	<0.0001	0.89 (0.82, 0.97)	0.0071
Low birth weight	0.95 (0.87, 1.04)	0.2550	0.92 (0.84, 1.01)	0.0687	0.93 (0.85, 1.02)	0.1219
Cesarean delivery	0.85 (0.81, 0.89)	<0.0001	0.85 (0.81, 0.89)	<0.0001	0.88 (0.83, 0.93)	<0.0001
Preterm birth	0.99 (0.91, 1.08)	0.8557	1.03 (0.95, 1.13)	0.4548	1.05 (0.96, 1.15)	0.2844

Serum iron was analyzed as a continuous variable, per 10 μmol/L increase. Adjusted I model was adjusted for age, BMI, and hemoglobin. Adjusted II model was adjusted for age, parity, BMI, systolic blood pressure, diastolic blood pressure, family history of hypertension, family history of diabetes, tobacco use, alcohol use, IVF, gestational week at testing, albumin, and hemoglobin.

### 3.3 Stratified analyses

The results of stratified analyses according to age, BMI, and parity are shown in Table 3. In the age-stratified analysis, among pregnant women aged <35 years, higher serum iron levels were significantly associated with a lower risk of PE, whereas no significant association was observed in women aged ≥35 years. Interaction analysis showed that there was no statistically significant interaction between age and the association between serum iron and PE. In the BMI-stratified analysis, among pregnant women with BMI < 24 kg/m^2^, higher serum iron levels remained significantly associated with a lower risk of PE, but not in those with BMI ≥ 24 kg/m^2^. Likewise, no statistically significant interaction was observed between BMI and serum iron. In the parity-stratified analysis, serum iron was significantly negatively associated with PE among primiparous women. Among multiparous women, this association was statistically significant in the unadjusted model and Adjust I model, while the association was not significant after full adjustment. Interaction analysis similarly showed no significant effect modification by parity on the association between serum iron and PE. The inverse association between serum iron and PE was more evident among women aged <35 years, those with BMI < 24 kg/m^2^, and primiparous women; however, the interaction tests for age, BMI, and parity were not statistically significant. The fitted curve illustrating the relationship between serum iron and hemoglobin is presented in Supplementary Figure S1.

**Table 3:** Subgroup analyses of the association between serum iron levels and preeclampsia.

Subgroup	Crude OR (95% CI)	*P*-value	Crude P for interaction	Adjusted I OR (95% CI)	*P*-value	Adjusted I P for interaction	Adjusted II OR (95% CI)	*P*-value	Adjusted II P for interaction
Age group			0.1678			0.5255			0.1354
<35 years	0.77 (0.71, 0.83)	<0.0001		0.83 (0.76, 0.91)	<0.0001		0.86 (0.79, 0.95)	0.0018	
≥35 years	0.86 (0.75, 1.00)	0.0455		0.88 (0.75, 1.03)	0.0996		0.99 (0.84, 1.17)	0.9225	
BMI group			0.9439			0.4044			0.1097
<24 kg/m^2^	0.88 (0.81, 0.97)	0.0068		0.82 (0.74, 0.90)	<0.0001		0.85 (0.77, 0.94)	0.0015	
≥24 kg/m^2^	0.89 (0.78, 1.01)	0.0642		0.88 (0.77, 1.01)	0.0633		0.98 (0.85, 1.13)	0.7711	
Parity group			0.7228			0.6141			0.8746
Primiparous	0.79 (0.73, 0.86)	<0.0001		0.86 (0.78, 0.94)	0.0008		0.89 (0.81, 0.97)	0.0101	
Multiparous	0.82 (0.69, 0.96)	0.0145		0.82 (0.68, 0.97)	0.0246		0.90 (0.76, 1.08)	0.2504	

Serum iron was analyzed as a continuous variable, per 10 μmol/L increase. Adjusted I model was adjusted for age, BMI, and hemoglobin. Adjusted II model was adjusted for age, parity, BMI, systolic blood pressure, diastolic blood pressure, family history of hypertension, family history of diabetes, tobacco use, alcohol use, IVF, gestational week at testing, albumin, and hemoglobin.

## 4 Discussion

### 4.1 Main findings

Based on a large cohort of 37,643 pregnant women, this study found that higher serum iron levels in early pregnancy were independently associated with a lower risk of PE. Serum iron levels were lower in the PE group than in the non-PE group, and smooth curve fitting showed that, after adjustment for age, BMI, and HGB, the risk of PE generally decreased as serum iron levels increased. Multivariable logistic regression further showed that for every 10 μmol/L increase in serum iron, the risk of PE decreased significantly in the non-adjusted model, the Adjust I model, and the Adjust II model. In addition to PE, higher serum iron levels were also associated with a lower risk of cesarean delivery, whereas no stable significant associations were observed with low birth weight or preterm birth. Stratified analyses showed that this inverse association was more evident among women aged <35 years, those with BMI < 24 kg/m^2^, and primiparous women, although the interaction tests were not statistically significant.

### 4.2 Comparison with previous studies

Previous studies on the relationship between iron status and PE have yielded inconsistent results (15–24). On the one hand, some studies based on samples collected at disease onset or during the second and third trimesters have suggested that women with PE have higher serum iron, ferritin, or hepcidin levels. Liu et al. reported in a meta-analysis that serum iron levels were higher in women with PE than in normal pregnant controls, although the included studies were predominantly case–control designs with late-pregnancy sampling (15). Bandyopadhyay et al. reported in a systematic review and meta-analysis that serum hepcidin levels may be elevated in women with PE, again based largely on samples obtained after disease onset (17). Mo et al. further showed in a longitudinal cohort study that higher maternal ferritin levels during pregnancy were associated with an increased risk of hypertensive disorders of pregnancy (23). Pyla et al. also observed higher iron levels in women with PE in a case–control study of multiple trace elements, although that study focused more on the overall association between multiple trace elements and PE severity and did not report OR values (24). These findings suggest that disturbances in iron metabolism or increased iron stores may be involved in the pathophysiology of PE during disease development (15, 17, 23, 24).

Other studies have suggested that lower serum iron in early pregnancy is associated with an increased risk of subsequent hypertensive disorders. Lewandowska et al. found in a prospective study that lower serum iron at 10–14 weeks of gestation was associated with an increased risk of subsequent gestational hypertension (18), a pattern consistent with our own results. Our study, which also assessed serum iron in the first trimester, demonstrated a graded inverse association between iron levels and subsequent PE risk. However, Ahmed et al. reported that among 60 women with PE and 60 healthy controls, with blood samples collected at the time of PE diagnosis (median gestational age, 38 weeks), no significant differences were found in serum iron status indices, hepcidin, or IL-6 levels (16). Jin et al. reported in a case–control study, with blood samples obtained before delivery (≥28 gestational weeks), that maternal iron status was associated with PE risk and placental iron transporter expression (19). Mendelian randomization studies have also shown inconsistent findings. Li et al. supported a possible causal association between serum iron and PE (20). By contrast, Yang et al. did not observe a clear genetic causal relationship (21), and the MR study by Rogne et al. did not provide conclusive evidence for a causal association between systemic iron status and pregnancy complications (22). Taken together, the current evidence supports the view that the relationship between iron status and PE is complex and is strongly influenced by the timing of measurement, the type of indicator used, and study design, rather than following a simple one-way pattern (16, 18–22).

Compared with the studies above, the main feature of the present study is its focus on serum iron in early pregnancy as a nutrition-related clinical laboratory indicator, and the use of a large cohort to assess its association with subsequent PE risk. Our findings are more consistent with the prospective observations of Lewandowska et al. (18), suggesting that lower serum iron in early pregnancy may indicate a higher risk of subsequent PE or hypertensive disorders of pregnancy. At the same time, our findings do not exclude the possibility that serum iron, ferritin, or hepcidin may increase during the second and third trimesters or after disease onset (15–17, 23, 24). These two sets of findings may reflect different stages and different dimensions of iron metabolism: early pregnancy may better reflect the relationship between maternal iron availability and the demands of placental development, whereas after disease onset, secondary disturbances in iron homeostasis related to inflammation, oxidative stress, and tissue injury may be more prominent (14, 23, 26).

### 4.3 Interpretation of the findings

#### 4.3.1 Research implications

From the perspective of nutrition-related laboratory indicators, serum iron is not only a commonly used marker of iron status, but may also reflect maternal nutritional reserve, placental adaptation, and maternal-fetal interface homeostasis (6–14). During pregnancy, maternal iron requirements increase substantially to support red blood cell expansion, placental development, and fetal growth (6, 10–14). The review and experimental work by Sangkhae et al. showed that iron homeostasis among the mother, placenta, and fetus is highly dynamic during pregnancy, and maternal iron status can directly influence placental and fetal iron distribution (13, 14). Therefore, if maternal iron availability is relatively insufficient in early pregnancy, it may theoretically affect normal placental formation and subsequent maternal vascular adaptation, thereby increasing the risk of PE (13, 14).

On the other hand, the relationship between iron and PE is unlikely to be a simple linear one. Previous studies have suggested that iron metabolism is also closely related to inflammation, oxidative stress, abnormal placental iron transport, and changes in the immune microenvironment (16, 17, 26). Zhong et al. emphasized in their review that iron-immune crosstalk at the maternal-fetal interface may contribute to the development of PE (26). Therefore, the finding in this study that “higher serum iron in early pregnancy was associated with a lower risk of PE” is more likely to reflect a link between insufficient maternal iron availability in early pregnancy and subsequent abnormal placental development, whereas the “elevated iron” observed in mid-to-late pregnancy or at disease onset may reflect secondary disturbances in iron metabolism during disease progression (15–17, 23, 26). This also suggests that in PE research, different iron status indicators and different sampling time points should not be interpreted as equivalent.

When interpreting the observed association, several additional physiological factors merit consideration. During normal pregnancy, plasma volume expands by more than 1 L on average compared with the non-pregnant state, exerting a substantial dilutional effect on circulating biomarkers, including serum iron. In pregnancies complicated by hypertensive disorders, preeclampsia, or fetal growth restriction, third-trimester plasma volume expansion is approximately 13.3% lower than that in normal pregnancy (27). This impaired expansion may reduce the dilutional effect and artifactually elevate measured serum iron concentrations, potentially obscuring an inverse association or even producing a spurious positive association in studies that sample blood after disease onset. This physiological phenomenon further reinforces the importance of early-pregnancy iron assessment, as plasma volume expansion is still limited during the first trimester, making early-pregnancy serum iron levels less confounded by these hemodynamic alterations.

#### 4.3.2 Clinical implications

Serum iron is a nutrition-related clinical laboratory indicator with potential clinical value. Compared with more complex or costly tests, serum iron is readily available, well established, and easily applicable in clinical practice. As a typical nutrition-related clinical laboratory indicator, serum iron not only reflects micronutrient status, but also, to some extent, reflects biological processes closely related to disease occurrence, including metabolism, inflammation, and maternal-fetal interface homeostasis. Rather than focusing solely on dietary intake, the present study emphasizes the use of objective laboratory indicators to identify high-risk populations, thereby providing a more practical approach for the clinical translation of nutritional exposures. In the risk management of obstetric and gynecologic diseases, especially PE, such an indicator may help with individualized management in early pregnancy when combined with maternal clinical characteristics (6–12). The stratified findings of this study suggest that the inverse association was more evident among women aged <35 years, those with BMI < 24 kg/m^2^, and primiparous women, indicating that serum iron may provide additional stratification information in women without prominent traditional high-risk factors. However, it should be emphasized that this study does not support the use of serum iron alone as a predictive tool for PE in clinical decision-making. Serum iron is affected by recent diet, iron supplementation, circadian rhythm, and inflammatory status, and a single indicator cannot fully reflect overall iron homeostasis (6–13). Therefore, to improve its value in precision medicine, a more reasonable approach would be to combine serum iron with hemoglobin, ferritin, transferrin saturation, inflammatory markers, and maternal clinical information, so as to improve the stability and interpretability of risk assessment (7–12). This study not only provides new nutrition-related laboratory evidence for PE as a representative obstetric disease, but also offers practical support for the application of nutritional indicators in disease prevention, risk control, individualized intervention, and long-term management.

### 4.4 Strengths and limitations

This study has several strengths. First, the large sample size provided high statistical power. Second, serum iron was measured in early pregnancy, allowing assessment of its association with subsequent PE risk, which is closely aligned with the clinical need for early identification and precision management of PE. Third, the study combined smooth curve fitting, stratified analyses, and interaction testing on the basis of multivariable models, making the results more complete and easier to interpret. At the same time, this study also has several limitations. First, as a retrospective observational study, it cannot establish strict causality. Second, this was a single-center study, and the generalizability of the findings still needs to be validated in different regions and populations. Third, the use of serum iron as the sole iron-status-related indicator represents an important limitation of this study, only serum iron was analyzed; ferritin, transferrin, total iron-binding capacity, hepcidin, inflammatory markers, and information on iron supplementation were not available, so iron homeostasis could not be comprehensively assessed, it cannot determine whether participants had iron deficiency, functional iron deficiency, or other iron metabolism abnormalities, nor can it propose or validate any serum iron threshold for identifying iron deficiency. Fourth, serum iron itself fluctuates considerably, and a single measurement may not fully represent iron status throughout early pregnancy. Fifth, although stratified analyses suggested a more evident inverse association in certain subgroups, the interaction tests were not statistically significant, and subgroup differences should therefore be interpreted with caution. Additionally, the study could not adjust for history of preeclampsia in previous pregnancies, family history of preeclampsia, or iron supplementation because this information was incompletely recorded in the electronic medical records. Residual confounding from these unmeasured factors may therefore exist. Future studies should systematically collect these clinical variables, and should also incorporate ferritin, transferrin saturation, total iron-binding capacity, inflammatory markers, and information on iron supplementation to more comprehensively and accurately assess iron status during pregnancy and its relationship with preeclampsia risk.

## 5 Conclusion

In summary, this study showed that higher serum iron levels in early pregnancy were significantly associated with a lower risk of PE. At the same time, the relationship between serum iron and PE is likely to be time-dependent and biologically complex, and a single indicator is insufficient to support direct clinical decision-making. Future studies should incorporate a more comprehensive iron status profile and prospective designs to further clarify the practical value of serum iron in the precision management of PE.

## Data Availability

The data that support the findings of this study are available from the corresponding author upon reasonable request.

## Supplementary material

Supplementary Figure 1 The fitting curve between serum iron and hemoglobin.
