# Supplementary figures and images for: Early-pregnancy serum iron as a nutrition-related clinical laboratory indicator for preeclampsia risk stratification: a retrospective cohort study
Source: Front Nutr. 2026 Jun 10;13:1853277. doi: 10.3389/fnut.2026.1853277 (PMC13290693; doi:10.3389/fnut.2026.1853277)

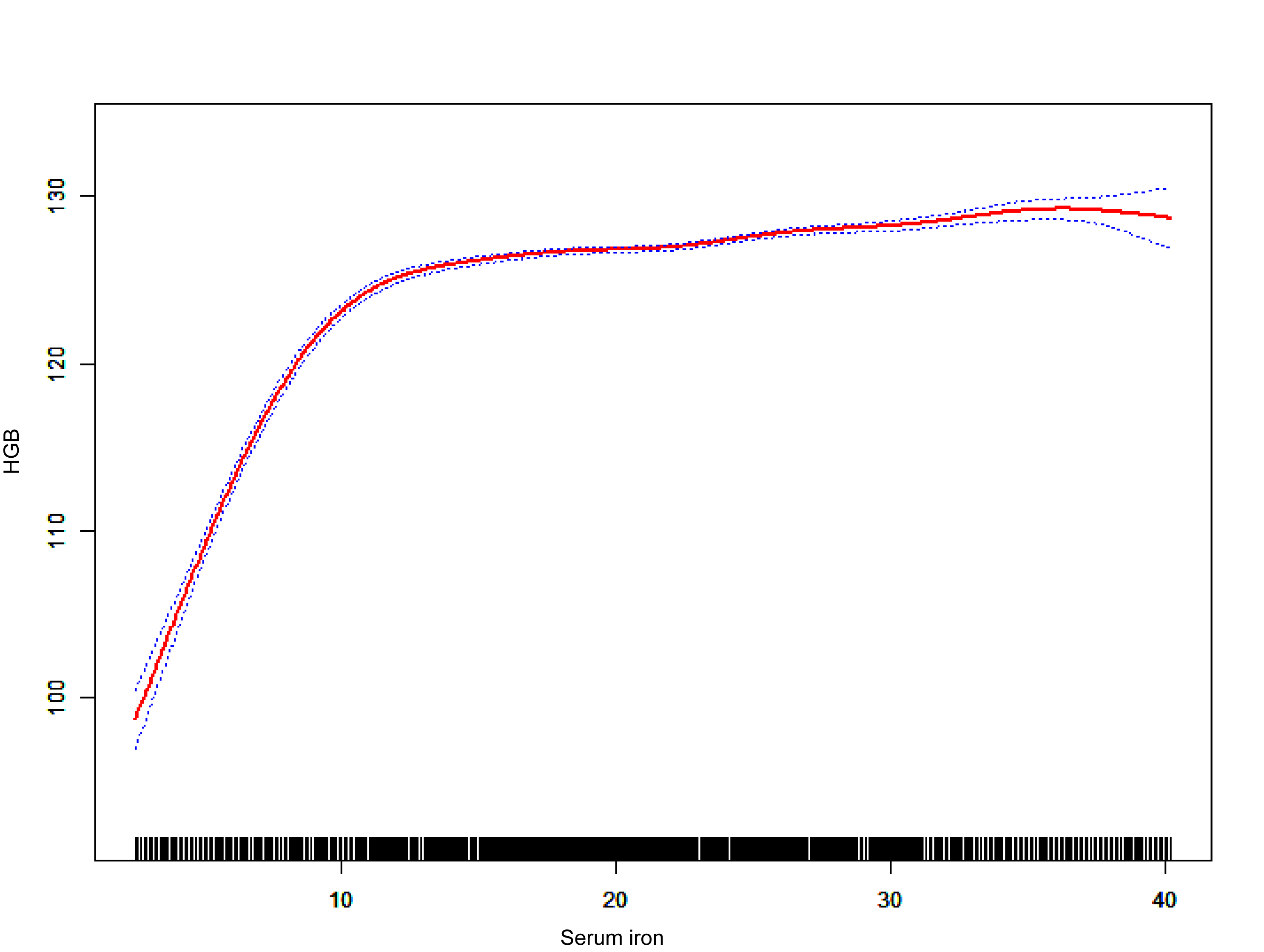

Supplement: Supplementary Figure 1 — The fitting curve between serum iron and hemoglobin. [file Figure_1.TIF]
